# JBrowse Connect: A server API to connect JBrowse instances and users

**DOI:** 10.1371/journal.pcbi.1007261

**Published:** 2020-08-18

**Authors:** Eric Yao, Robert Buels, Lincoln Stein, Taner Z. Sen, Ian Holmes

**Affiliations:** 1 Department of Bioengineering, Stanley Hall, University of California, Berkeley, California, United States of America; 2 U.S. Department of Agriculture, Agricultural Research Service, Western Regional Research Center, Crop Improvement and Genetics Research Unit, Albany, California, United States of America; 3 Ontario Institute for Cancer Research, Toronto, Ontario, Canada; University of Technology Sydney, AUSTRALIA

## Abstract

We describe JBrowse Connect, an optional expansion to the JBrowse genome browser, targeted at developers. JBrowse Connect allows live messaging, notifications for new annotation tracks, heavy-duty analyses initiated by the user from within the browser, and other dynamic features. We present example applications of JBrowse Connect that allow users 1) to specify and execute BLAST searches by either running on the same host as the webserver, with a self-contained BLAST module leveraging NCBI Blast+ commands, or via a managed Galaxy instance that can optionally run on a different host, and 2) to run the primer design service Primer3. JBrowse Connect allows users to track job progress and view results in the context of the browser. The software is available under a choice of open source licenses including LGPL and the Artistic License.

This is a *PLOS Computational Biology* Software paper.

## Introduction

JBrowse [1] is a dynamic HTML genome browser installed at over 3,000 sites with over 40,000 monthly active users (Google Analytics, May 2019) and a thriving ecosystem of over 50 plugins, many contributed by third parties unaffiliated directly with the project (https://gmod.github.io/jbrowse-registry/). As one of the first JavaScript client genome browsers with a web API that can work directly from a static set of files, JBrowse can be run using any web server, including very lightweight web servers such as NGINX, Node.js Express, or even PublicFile.

By design, similar to all “serverless” or “static” applications, the communication of information in JBrowse is one-way; there is no concept of a session or persistent connection, and no means for clients to push messages back to the server, or to other peer clients. For many purposes, this static model can be advantageous. For example, it is easy to deploy a genome as a “static site,” i.e., a set of files that are simply served up with no processing by a web server, and so can reside in cheap storage, e.g. in an Amazon S3 bucket. The static model is also inherently secure, as no code needs to be run on the server after the initial deployment. Thus, for many applications, a client-only genome browser is ideal. However, if the genome browser is intended to be not just a vehicle for displaying information, but a portal to initiate analysis, then it can be limiting that the client and server have no means for bidirectional communication during a browsing session. Many clinical and scientific collaborations of the future will take place either within the genome browser itself, or with genomes as an essential part of the view; so that the primary evidence supporting gene models, variant calls, pathway-level analyses, engineered genome sequences, or other genomics constructs can be shared, annotated, filed, and reviewed. Further, since a genome browser is a core part of many commercial “bioinformatics workbench” applications such as products from Geneious [2] and Qiagen [3], offering functionality that goes beyond visualization such as primer design and homology searches, there is a clear demand for free, publicly-available academic equivalents to offer similar services. In order to build the rich interactive dashboards that these applications will require, some form of server is likely to be necessary.

As a partial workaround for these limitations, many groups have integrated some kind of server-side processing into JBrowse; either by having it be the last step of a workflow that generates and indexes static files, or by integrating it more deeply into a content management system. Some of these integrations are reusable in general contexts. Notably, the Apollo project enables biocurators to collaborate in real time editing genome annotations [4,5]. Bioinformatics web applications such as Tripal [6] and Galaxy [7] have extensions that integrate JBrowse to some extent. JBrowse’s plugin framework facilitates this sort of customization. However, since JBrowse was originally designed as a client-side application, with no API for dynamic server integration, there are limits to how smoothly such extensions can be integrated with the JBrowse user interface. Apollo, for example, includes a BLAST button but this is not deeply integrated with JBrowse. In general, JBrowse offers no means to track job progress from within the genome browser, and no mechanism for asynchronous notifications when new updates and results are available. Galaxy allows for general analysis, and can construct a JBrowse instance as a product of that analysis, but has the same limitations on analyses to be initiated from within the browser itself: the genome browser itself is oblivious to the work being done. All of these mechanisms must be implemented from scratch in each case, with no common infrastructure within JBrowse that they could leverage.

In this paper, we present JBrowse Connect, an optional and generic server extension to JBrowse that consists of two components: one that runs together with JBrowse on the client web browser, and the other that runs as an extensible server back-end together with the analysis tools. JBrowse Connect is designed to serve client requests for management and analysis of genomic data; to broker messaging between client and server (for example, to notify logged-in users of newly-added annotation tracks); to enable integration with 3rd-party analysis tools; and to provide mechanisms for straightforward application-specific extensions called “hooks” on both client and server. It leverages Sails (https://sailsjs.com), an open source JavaScript model-view-controller framework with object-relational business logic and integration with WebSockets, a modern duplex communication protocol for use in web applications.

Like the JBrowse client, JBrowse Connect is extensible: it is straightforward to develop new services and workflows as plugin “hooks” that can be exposed to users within the browser, and since these plugins are modular, they can be reusably shared with other developers. As an illustration of how this extensibility works, we have developed two plugins. The first plugin presents a front end for initiating a BLAST search in several alternate ways [8]. The other plugin presents an interface to the Primer3 tool for primer design [9,10]. To our intended audience of developers, these plugins demonstrate the API for developing a JBrowse plugin that triggers, monitors, and then collects the results of an analysis workflow. These plugins can run locally i.e. on the same computer as the JBrowse Connect server itself, or can optionally hosted by a workflow manager such as Galaxy either on the same server, on a compute cluster, or on the cloud. The plugins are also functional from the user’s perspective, demonstrating the experience of initiating a nontrivial analysis from within JBrowse. However, in deployment they would typically require customization to the specific needs and user account management infrastructure of the hosting institution.

As noted above, this manuscript is primarily targeted at developers interested in using JBrowse Connect to build server capabilities, particularly analysis workflows, into JBrowse. As a working example, we describe the two JBrowse Connect plugins we have developed for BLAST and Primer3 analysis. In the section titled “JBrowse Connect from the user’s perspective”, we first describe how end-users can interact with these plugins, starting with general outlines and then proceeding to a more concrete user story involving the GrainGenes [11] website (https://wheat.pw.usda.gov). In the subsequent section, “JBrowse Connect from the developer’s perspective”, we outline the technical architecture of JBConnect (the core server software for JBrowse Connect) and JBClient (the corresponding JBrowse client plugin), and discuss how JBrowse Connect can be extended to implement novel, bespoke workflows.

## Design and implementation

### JBrowse Connect from the user’s perspective

In this section we describe the JBlast plugin,which includes significant additional functionality for dynamic filtration of BLAST hits, and the Primer3 plugin. We start from the perspective of the end user, and then discuss the underlying interactions and component architecture that are more relevant to the developer seeking to add their own workflows.

#### JBlast from the user’s perspective

A JBrowse Connect instance—which by default includes the JBlast plugin—looks mostly identical to vanilla JBrowse, but new buttons appear in the user interface. Using these buttons, the user can select a region of the reference genome from within the genome browser and, with a single mouse click, dispatch it for search against an instance-specific BLAST database. The user may alternatively submit a query sequence and BLAST it against the reference genome; these two alternative models are referred to as “reference-as-query” and “reference-as-target”. The progress of the BLAST search may be tracked through a popup queue window from within the user’s JBrowse instance. When the results are available, they show up immediately in the user’s web browser as a new track. Illustrating that JBrowse Connect plugins can provide new functionalities on both the server and the client, the JBlast plugin includes a client-side plugin that allows the results of a BLAST search to be dynamically filtered.

**Fig 1:**
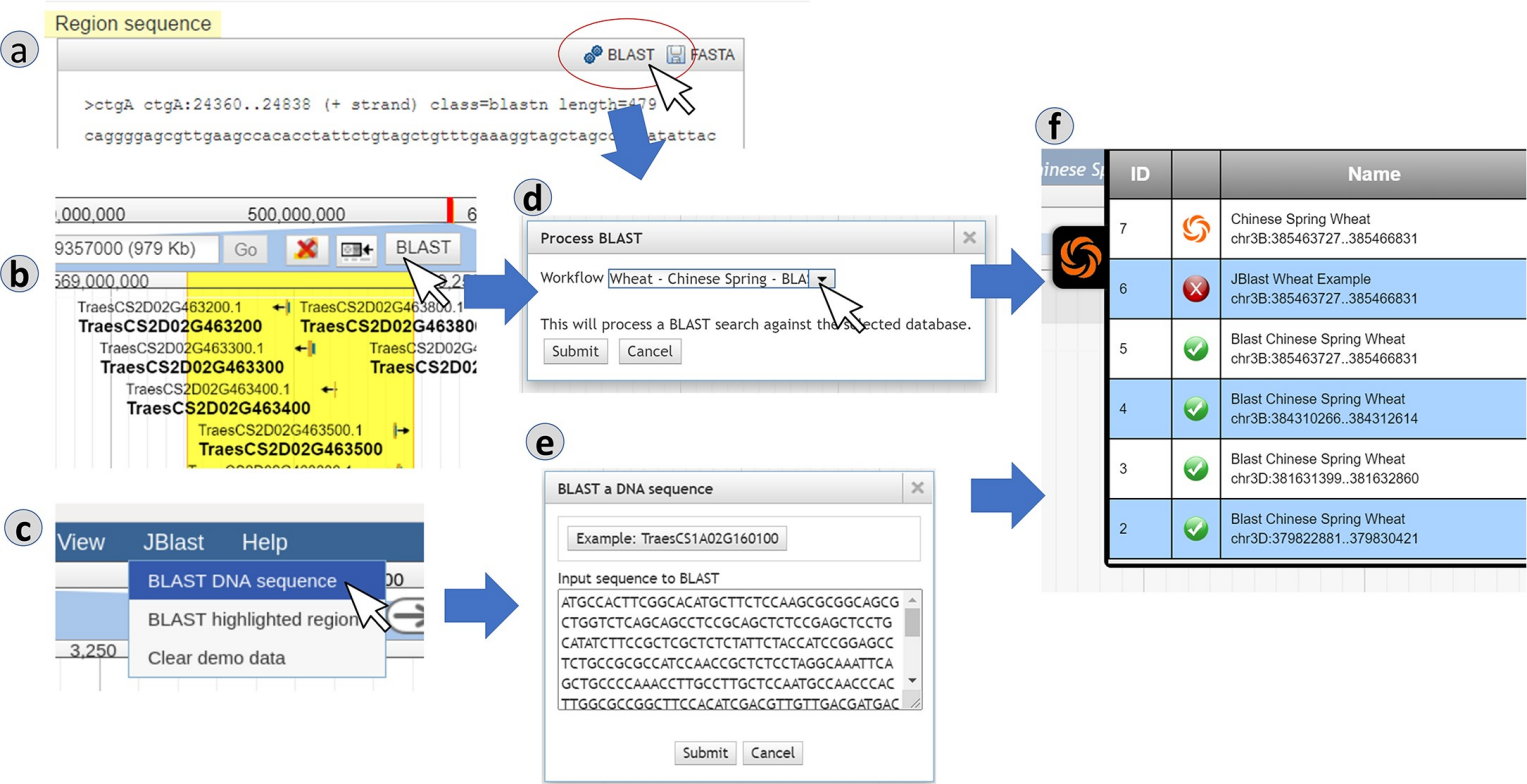
A sequence can be submitted for BLAST search in one of several ways, either with reference-as-query (a region of the genome is selected and then BLASTed against a built-in database) or with reference-as-target (a sequence is uploaded by the user and then searched against the genome). After confirming, the user can track the progress of the search in the built-in job queue. Panels: (a) selection by feature, (b) selection by highlighting, (c) selection by menu, (d) BLAST dialog for reference-as-query, (e) BLAST dialog for reference-as-target, (f) job queue.

Fig 1 shows the different ways of submitting a BLAST query using a real-world example. The tracks were taken from the GrainGenes Genome Browser page (https://wheat.pw.usda.gov/jb/?data=/ggds/whe-iwgsc2018) for the Chinese Spring IWGSC RefSeq v1.0 wheat genome assembly [12]. The first method of initiating a BLAST search (1a) is to select an existing feature from an existing track. In the feature’s Details dialog box under the Region sequence a BLAST button is available; clicking this button will start the BLAST analysis. The second method of initiating a BLAST search (1b) is to use JBrowse’s highlight feature to select a region of genomic sequence. Upon highlighting a region, a BLAST button will appear on the toolbar. An equivalent option to “BLAST highlighted region” is also accessible from the JBlast menu, as shown in 1c. Initiating the BLAST analysis via any of these methods leads to the Process BLAST dialog (1d) which allows the user to choose the Workflow that will execute the BLAST operation. A third method (1c) is accessed from the JBlast menu, “BLAST DNA Sequence.” Selecting this item leads to the Blast a DNA Sequence dialog, which allows for entering an arbitrary DNA sequence by pasting sequence. Upon submitting the job, a new job item will appear in the Job Queue panel (1f).

**Fig 2:**
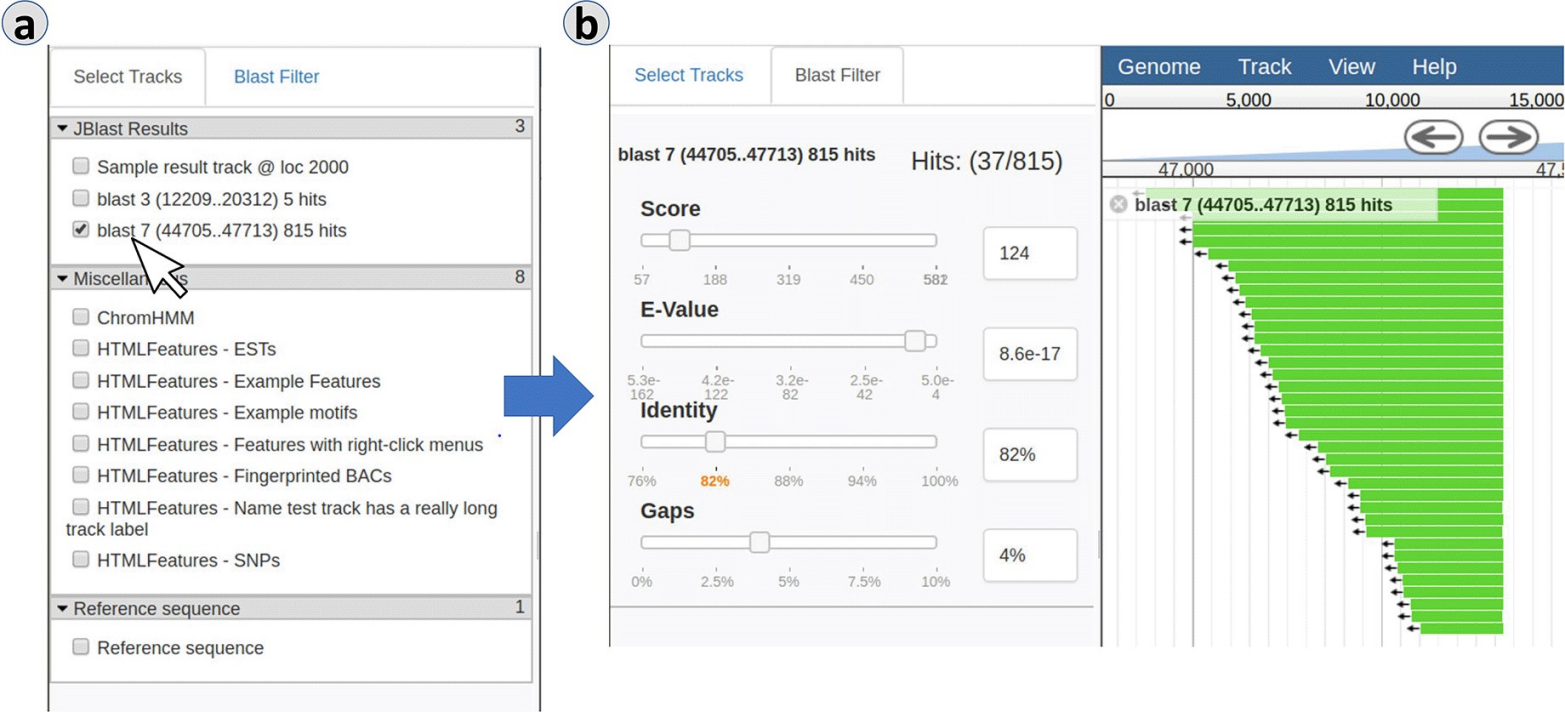
Results from the BLAST search can be dynamically filtered. In the reference-as-query mode, the results are displayed as features that are all located in the region of the genome that was selected by the user as the query region. Panels: (a) BLAST result tracks, (b) BLAST filter.

Fig 2 shows JBlast query results that were initiated in Fig 1 in reference-as-query mode where results are mapped to the source sequence and all hit results are stacked together within the query region. If BLAST is successful and there are hits, a new result track will appear in the track selector (2a). When the user selects the track, it will appear in the main view, accompanied by a BLAST Filter panel (2b) that allows the user to interactively and dynamically filter results by score, E-value, sequence identity, or alignment gappiness.

**Fig 3:**
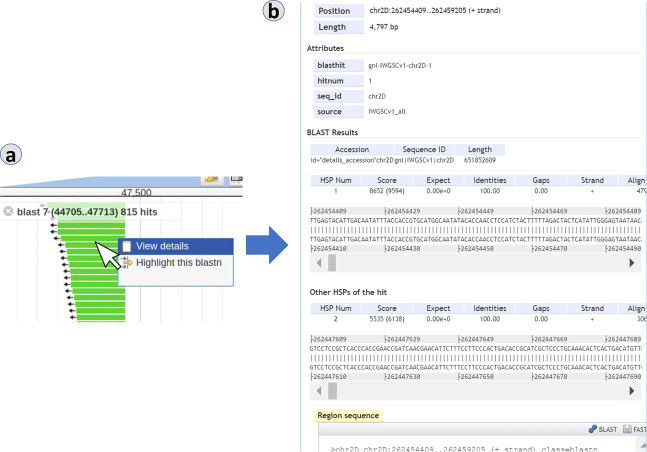
Details of a BLAST hit appear in a feature popup and include location, alignment, and external database links. Panels: (a) BLAST hit features, (b) BLAST hit details.

In Fig 3, the features in a BLAST result track (3a) are BLAST hits, the details of which can be viewed in the feature details dialog box (3b) by double clicking the feature or access via its right-click menu. The High-Scoring Segment Pair (HSP) is provided for the given hit along with various other hit details, as well as other HSPs from the same hit.

**Fig 4:**
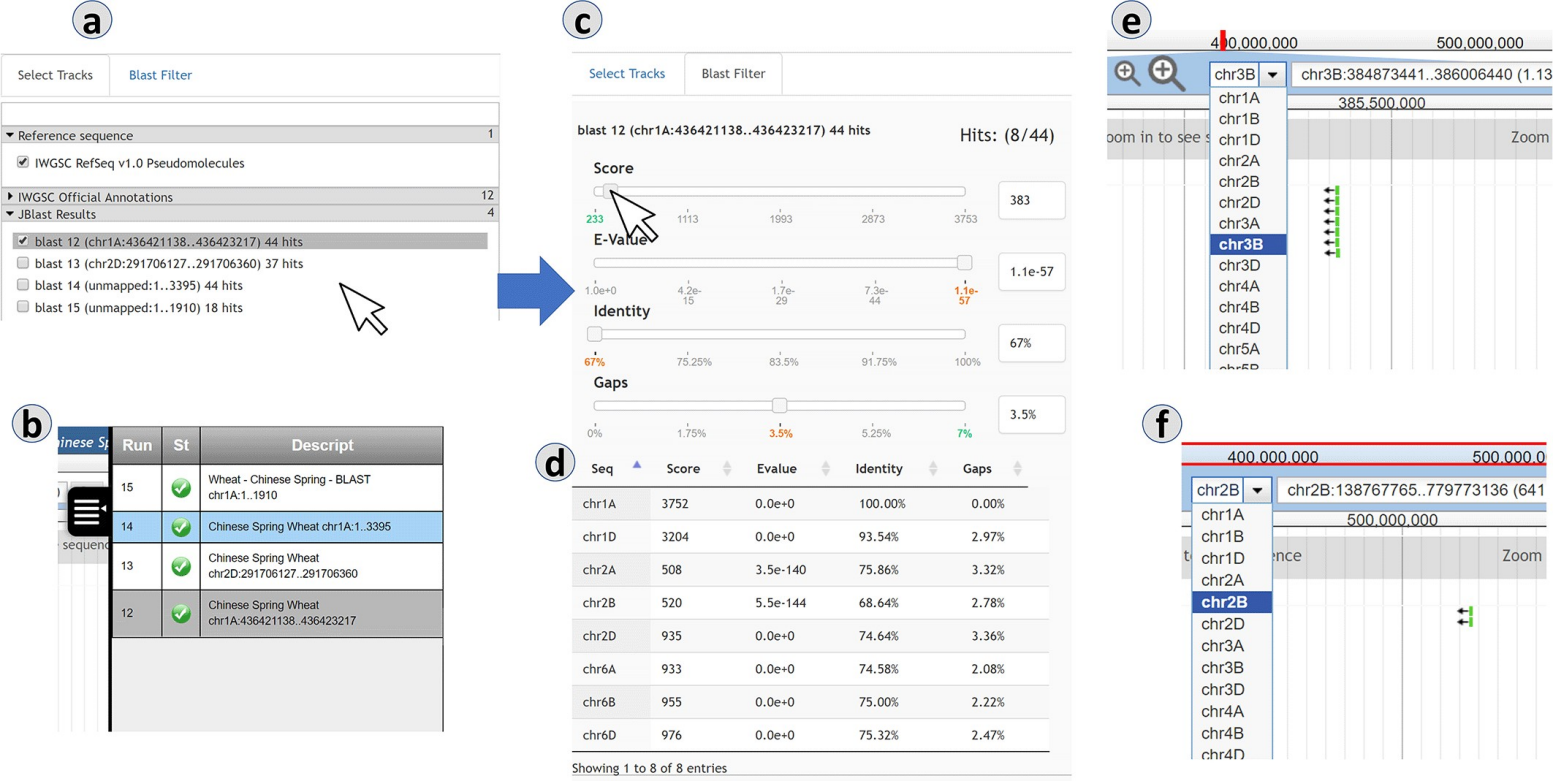
In the reference-as-target mode, matches to the user-uploaded sequence may be located all over the genome. As with the reference-as-query mode, the BLAST hits can be dynamically filtered through the control panel. Panels: (a) BLAST result tracks, (b) completed jobs in queue, (c) BLAST filter, (d) BLAST results table, (e/f) BLAST result features.

In Fig 4, analysis of BLAST search made using the online JBlast demo at GrainGenes, which is configured to use Chinese Spring Wheat as the reference genome. In this case, the BLAST database used is Chinese Spring itself. Here, we render the results in reference-as-target mode. Since hit results will all be within Chinese Spring, the hit results will be dispersed within the reference sequence and chromosomes.

In the reference-as-target rendering, below the filter sliders (4c), a result table (4d) is present to aid in navigating to the hits of interest. In this example, 4e and 4f show results from the same BLAST in two different regions/chromosomes.

#### BLAST processing

**Fig 5:**
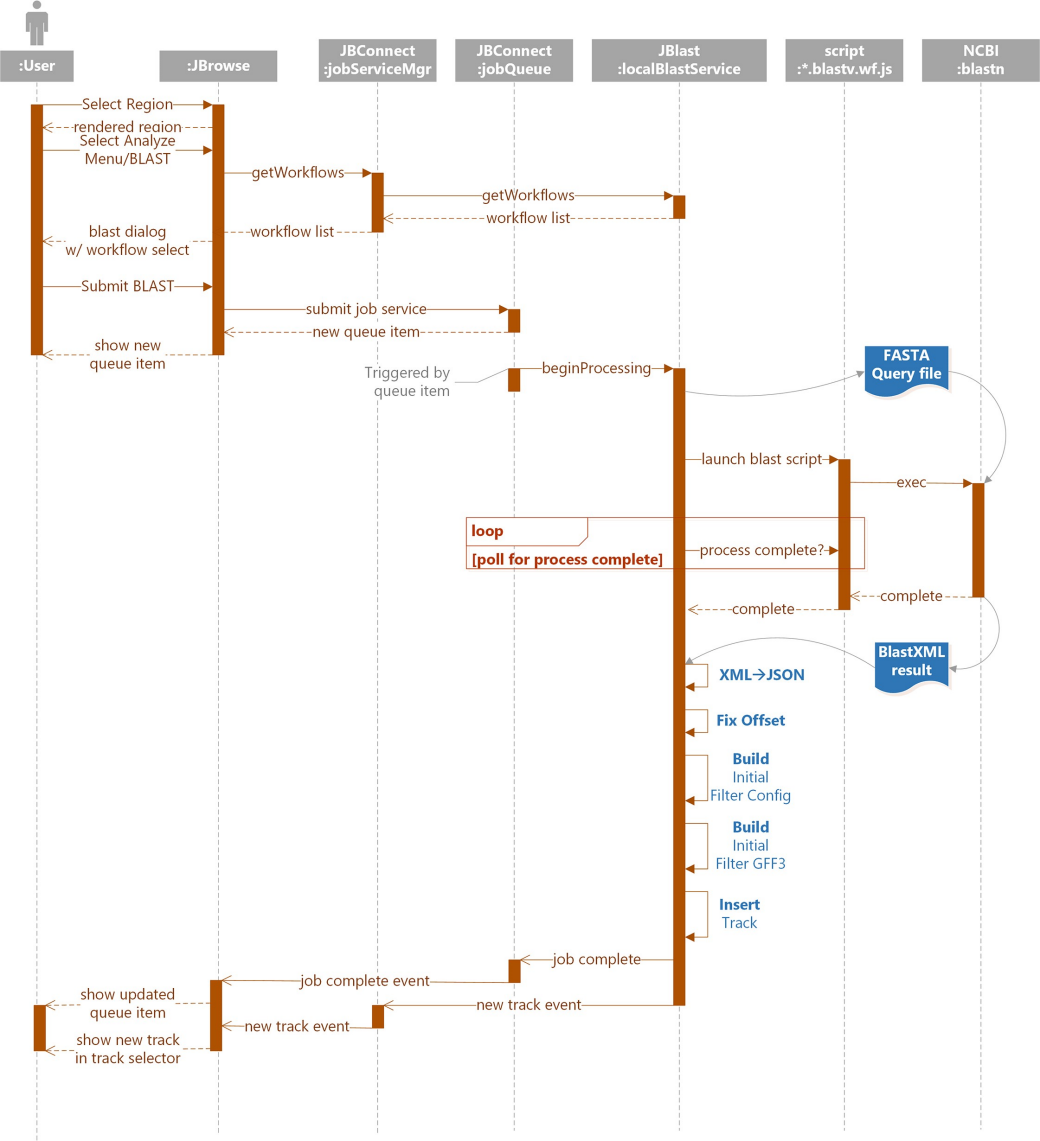
Interactions for the standalone BLAST workflow flow from the user, via the JBrowse client, through the JBConnect server, and into the JBlast module, which then runs NCBI BLAST locally on the server, processes and packages the results, and returns them to the user.

The interaction diagram in Fig 5 presents a more detailed view of the communication between JBrowse client and various modules within the JBConnect and, in this case, the stand-alone BLASTn module. Galaxy BLAST and Primer3 module interactions are discussed later.

The primary components in Fig 5 are:

- JBrowse is the JavaScript genome browser.
- JBConnect which consists of
  ○ jobService control provides the primary interface to the JBlast:localBlastService
  ○ Queue hosts the job queue API
  ○ JBlast:localBlastService manages the life-cycle of a local BLAST job.
  ○ *.blastv.wf.js represents the local workflow script. The script that runs is chosen by the user in the workflow selection interface.
  ○ Blastn is the NCBI blastn command.

The processing of the standalone BLAST job service (localBlastService), as shown in Fig 5.

The interaction diagram in Fig 5 describes the communication between various modules for processing query sequences of the Standalone BLAST service.

1. The job submission cycle begins with the user selecting the desired region by highlighting the region with JBrowse. This becomes the query sequence. (See Fig 1B)
2. User selects the BLAST highlighted region option from the Analyze menu. (See Fig 1C).
  - JBrowse calls getWorkflow from jobServiceMgr,
  - Which in turn calls getWorkflow from localBlastService,
  - The Process BLAST dialog then appears. (See Fig 1D) If there is only one workflow, it will automatically be selected and the workflow selection option will not appear.
3. The user selects Submit (Fig 1D).
4. A job structure and the query sequence are submitted to the jobQueue.
5. The job queue panel (Fig 1F) will appear showing the submitted job. This completes the job submission cycle.
6. A monitor in the job queue detects the queue item then calls beginProcessing in localBlastService. This triggers localBlastService’s execution phase:
  - The localBlastService begins by generating a FASTA file as a query sequence.
  - The *.blastv.wf.js workflow script is launched, which in turn, launches blastn command.
  - Upon completion, localBlastService acquires the resulting BLAST XML file from blastn.
  - The XML is converted to JSON
  - Offsets are adjusted for the resulting hits
  - It builds an initial filter config file for the resulting hits
  - It builds an initial GFF3 file
  - It inserts the newly created GFF3 as a track in JBrowse
7. localBlastService finally notifies the client of the new track and completed job.

In principle JBConnect can use a variety of back-end bioinformatics workflow processors. Currently implemented options include 1) simply running software such as BLAST or Primer3 on the same server as JBConnect itself, or 2) using a Galaxy workflow to perform the BLAST searches. JBrowse Connect plugins can talk to other back-end bioinformatics services, including scientific computation providers such as Cyverse. They can also act as simple proxies for other queuing systems.

#### Primer design from the user’s perspective

Primer3 is a widely-used software for designing unique primer sequences avoiding both (a) similarity to other regions of the genome and (b) strong RNA secondary structure [9,10]. This software is commonly available in genomics workbench suites; for example, it has previously been made available as a GBrowse plugin [13]. We implemented a Primer3 hook as an alternate demonstration of a JBConnect hook. It is simpler than the JBlast hook, in that it is lacking the extended filtering interface for results. The Primer3 hook includes a workflow service implemented to accept and process Primer3 results and a JBrowse plugin that allows the user to select and then perform an analysis on a region of the genome. The results are rendered as a feature track, the features being the candidate primer sequences recommended by Primer3.

**Fig 6:**
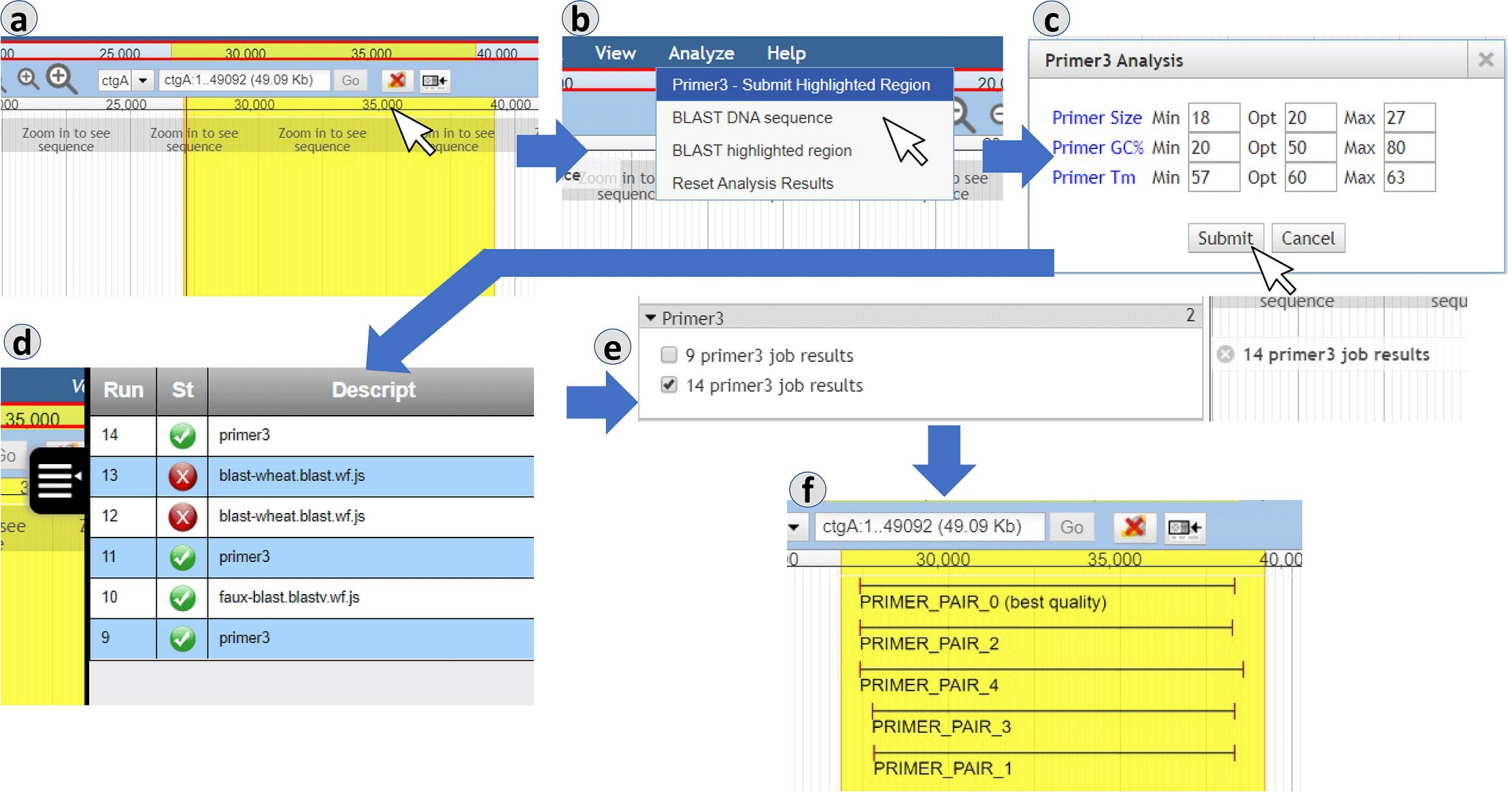
The Primer3 user interface involves selecting a region, then submitting it for processing. The job can be tracked using the built-in queue view. The returned primer pairs are displayed as genome features. Panels: (a) selection by highlighting, (b) running Primer3 via menu, (c) Primer3 analysis dialog, (d) job queue, (e) Primer3 results tracks, (f) Primer3 results.

Fig 6 demonstrates the process of submitting a query sequence for Primer3 analysis. The user starts by highlighting the region of interest using the highlight feature of JBrowse (6a). The user then chooses Primer3 from the Analyze menu (6b) which opens the Primer3 Analysis dialog (6c). From here, the primer parameters can be modified prior to submission. When submit is selected, the query sequence and Primer3 parameters are passed to the server. Upon successful completion (6d), the resulting track will appear in the track selector (6e). When the track is selected, the resulting primer pairs will be displayed (6f).

**Fig 7:**
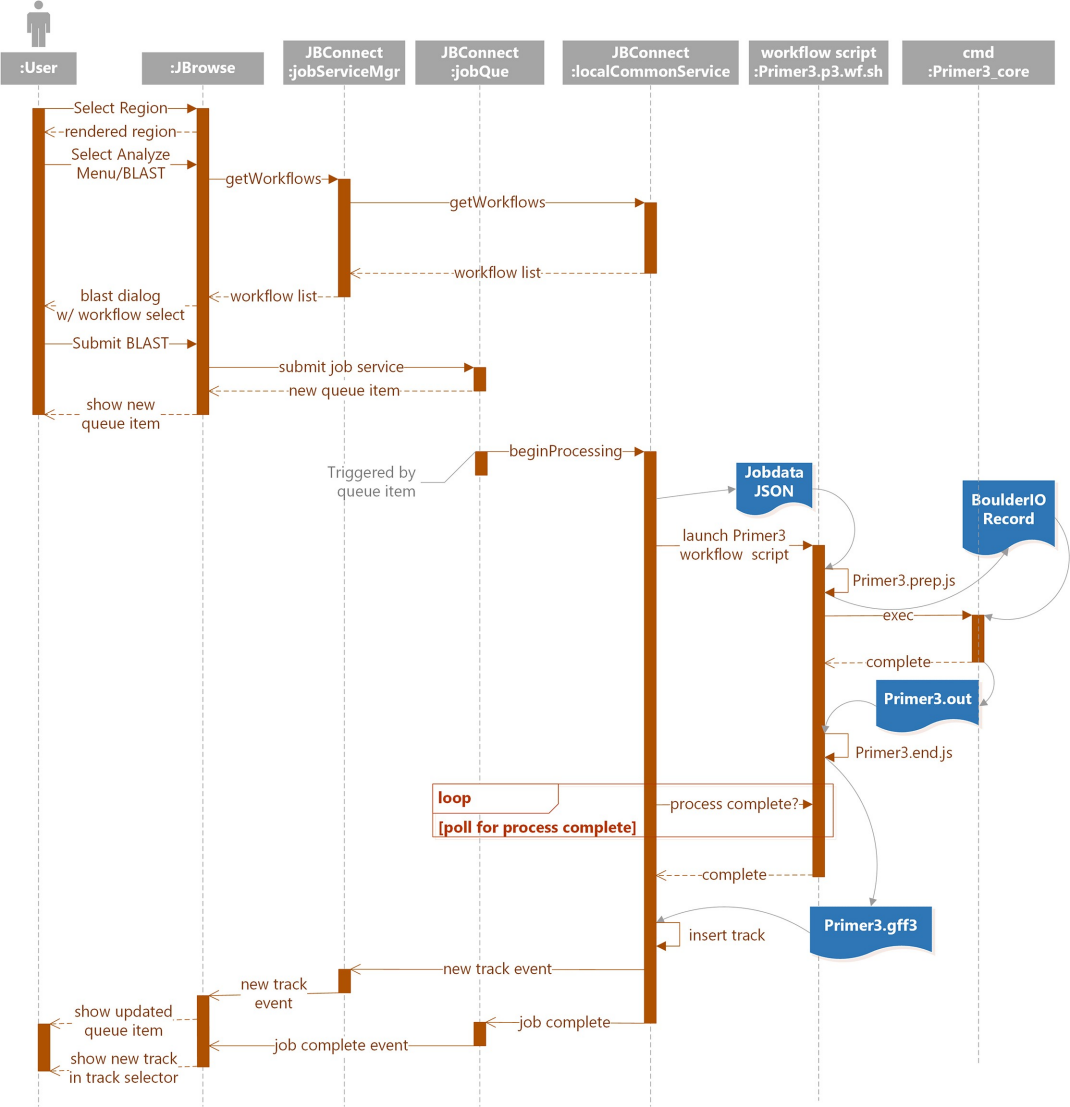
Interactions between components during a Primer3 job involve the user and JBrowse client, the JBConnect server, and a Primer3-specific script that wraps the Primer3 executable itself.

The interaction diagram in Fig 7 describes the communication between various modules for processing query sequences with Primer3.

1. The job submission cycle of Fig 7 begins with the selection of a highlighted region, the user interface flow of which is shown in Fig 6A. This becomes the query sequence.
2. User selects the Primer3 option from the Analyze menu. (See Fig 6B).
  - JBrowse calls getWorkflow from jobServiceMgr,
  - Which in turn calls getWorkflow from localCommonService,
  - Which acquires the available workflow list
  - The Primer3 Analysis dialog then appears. (See Fig 6C) If there is only one workflow, it will automatically be selected and the workflow selection option will not appear.
3. User selects Submit (Fig 6C).
4. A job structure and the query sequence are submitted to the jobQueue.
5. The job queue panel (Fig 6D) will appear showing the submitted job. This completes the job submission cycle.
6. A monitor in the job queue detects the queue item then calls beginProcessing in localCommonService. This triggers localCommonService’s execution phase:
  - The localCommonService begins by generating a job data file JSON file that contains submission information and the query sequence.
  - The Primer3 workflow script is launched and passed the job data file.
  - The Primer3.prep.sh script is launched and generates the BoulderIO record, the Primer3 configuration file.
  - Primer3_core is then executed with BoulderIO record.
  - Upon completion of Primer3, a Primer3.out file is generated and passed to Primer3.end.ps.
  - Primer3.gff3 file is then generated and passed to localCommonService for track insertion into JBrowse.
7. WorkflowService finally notifies the client of the new track and completed job.

### JBrowse Connect from the developer’s perspective

#### JBConnect hosts an instance of JBrowse extending it with analysis capability

JBConnect is a web service that hosts an instance of JBrowse with extended analysis capabilities through a RESTful API. Among other functions, JBConnect can inject additional plugins into the libraries that constitute the JBrowse client; one such injected JBrowse plugin is JBClient (bundled with JBConnect), which augments JBrowse with a login panel and a Job Queue panel (Fig 1F), a clean and simple user interface for viewing queued, completed, active, and failed jobs. It illustrates a straightforward use of JBConnect’s queue as a simple interface to more powerful workflow engines such as Galaxy, which in turn connects to many kinds of cluster- and cloud-based workflow management systems.

#### JBConnect uses Sails.js as a Model-View-Controller framework

JBConnect is a Sails.js application (https://sailsjs.com). As such, it requires Node.js as the JavaScript runtime engine, and further requires that the Node.js module Express is installed as the webserver. Sails.js has a number of features, standard for a web application framework, which are used by JBConnect and may readily be extended by developers; these include a model-view-controller architecture, a built-in object-relational mapping with multiple back-end database handlers (Waterline ORM), a WebSockets API (Socket.io) with publish-subscribe support for handling messaging and notifications between client and server, a flexible authentication service (Passport.js), and a framework called Blueprints which ties models, controllers, database objects into a consistent server API and REST interfaces between client and server, providing notification to clients of database updates.

**Fig 8:**
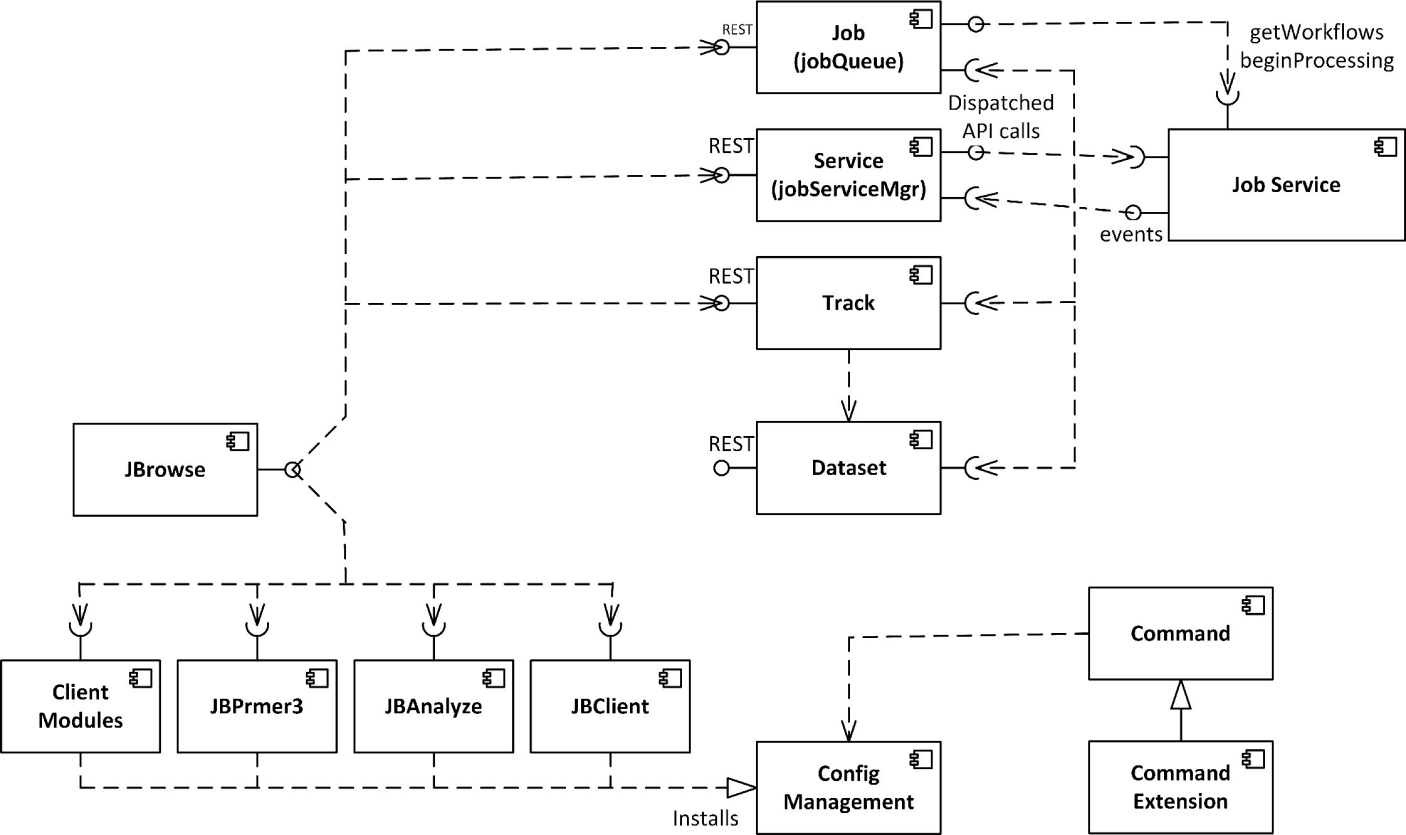
The JBConnect server architecture encompasses plugins (including JBPrimer3, JBAnalyze, JBClient), various Models (including Job, Service, Track, & Dataset), any number of Job Services, and various utility modules.

Fig 8 describes the architecture of JBConnect server. The following describes each component:

1. Models / Controllers—All the models have their APIs for internal access as well as REST APIs for external access.
  - The Job (jobQueue) provides the interface for submitting jobs and managing the queue. It monitors job service progress and fires events when there are state changes. Basic CRUD operations are available for jobs.
  - The Service model (aka jobServiceMgr) manages job services. The system uses it to register job services such as galaxyBlastService, localBlastService.and localCommonService (used by Primer3). It provides a dispatch interface to the job services which perform the analysis work. The client side plugins communicate to the job services through the jobServiceMgr module.
  - The Track model encapsulates the JBrowse tracks. It synchronizes model tracks with JBrowse tracks. CRUD operations are available for the Track model and changes are automatically pushed to JBrowse’s track list configuration file.
  - The Dataset model represents JBrowse datasets. Typically a dataset may represent an organism and its collection of tracks. The Dataset model synchronizes with datasets that are configured at startup, but CRUD operations are not available.
2. Job Service represents a job service (localBlastService, galaxyBlastService, or localCommonService) that performs some analysis work and manages the lifecycle of analysis operations. It also serves as an adapter to third party APIs. Job services may also host APIs that have a REST interface and provide a mechanism to access its functions through a /service/exec REST dispatched API.
3. JBrowse Plugins—two JBrowse plugins (JBClient, JBAnalyze) are JBrowse client components to JBConnect.
  - JBClient manages login/logout GUI interface in JBrowse. It handles track insertion and update events from the client and renders them in the track selector. It also handles Queue events and renders jobs in the job queue panel.
  - JBAnalyze implements the Analyze Menu and provides a configuration interface that can be used by other plugins to customize Analyze menu items and analysis dialog boxes. It is used by JBlast and JBPrimer3.
  - JBPrimer3 is part of the primer3-jbconnect-hook that is an included JBConnect hook module to the JBConnect package. It provides the menu interfaces and dialog boxes to handle Primer3 job submissions.
  - Client modules are modules like jQuery-UI, which other JBrowse plugins are dependent on.
  - Plugins and dependency modules are pushed to the JBrowse configuration by the Configuration Management module. The Config module also aggregates plugins and modules from JBConnect Hooks (such as the primer3-jbconnect-hook) as well as aggregates config parameters.
4. Command is the jbutil command used for diagnostic and configuration.
5. JBConnect hooks can extend the command set through its own command extension. Implementation. The system automatically detects this if it exists in a hook.

#### JBConnect object-relational model

The core database tables of JBConnect, termed “model objects” in Sails’ object-relational mapping, represent the data structures required to perform asynchronous remote analysis in a genome browser. Sails is extremely flexible as to the particular database back-end that is used, and the object-relational mapping is an abstraction that enables this flexibility; by default, JBConnect is configured to use JSON database.

Two of these model objects, “Dataset” and “Track”, represent core concepts of the JBrowse data model [1]. A Dataset, which typically corresponds to an individual genome, consists of a set of reference sequences and associated Tracks, which are annotations that may be displayed in the browser. When these entities are manipulated through JBConnect’s API, the static files that the JBrowse uses as indices of tracks and datasets are automatically updated, and notifications are sent to all connected JBrowse clients that the tracks or datasets have been changed and should be refreshed.

The “Job” model object encapsulates analysis jobs. Using the JBConnect API, jobs may be submitted, listed, updated, or destroyed. The JBClient plugin uses this API to manage jobs. Two other new model objects, “Passport” and “User”, provide a flexible interface to local authentication mechanisms and user accounts.

Some user isolation is provided for tracks generated and jobs by JBConnect such that one user cannot see the tracks and jobs of another user. This is done for illustrative purposes and is not intended to be highly secure.

#### JBConnect offers an extensible framework for plugins (“hooks”)

JBConnect Hooks framework leverages Sails Installable Hooks framework and extends it in a number of ways, providing a mechanism for expanding JBConnect’s functionality using NPM modules. A developer seeking to extend JBConnect should not need to modify this architecture, but it is useful to understand its general layout in order to navigate the process.

**Fig 9:**
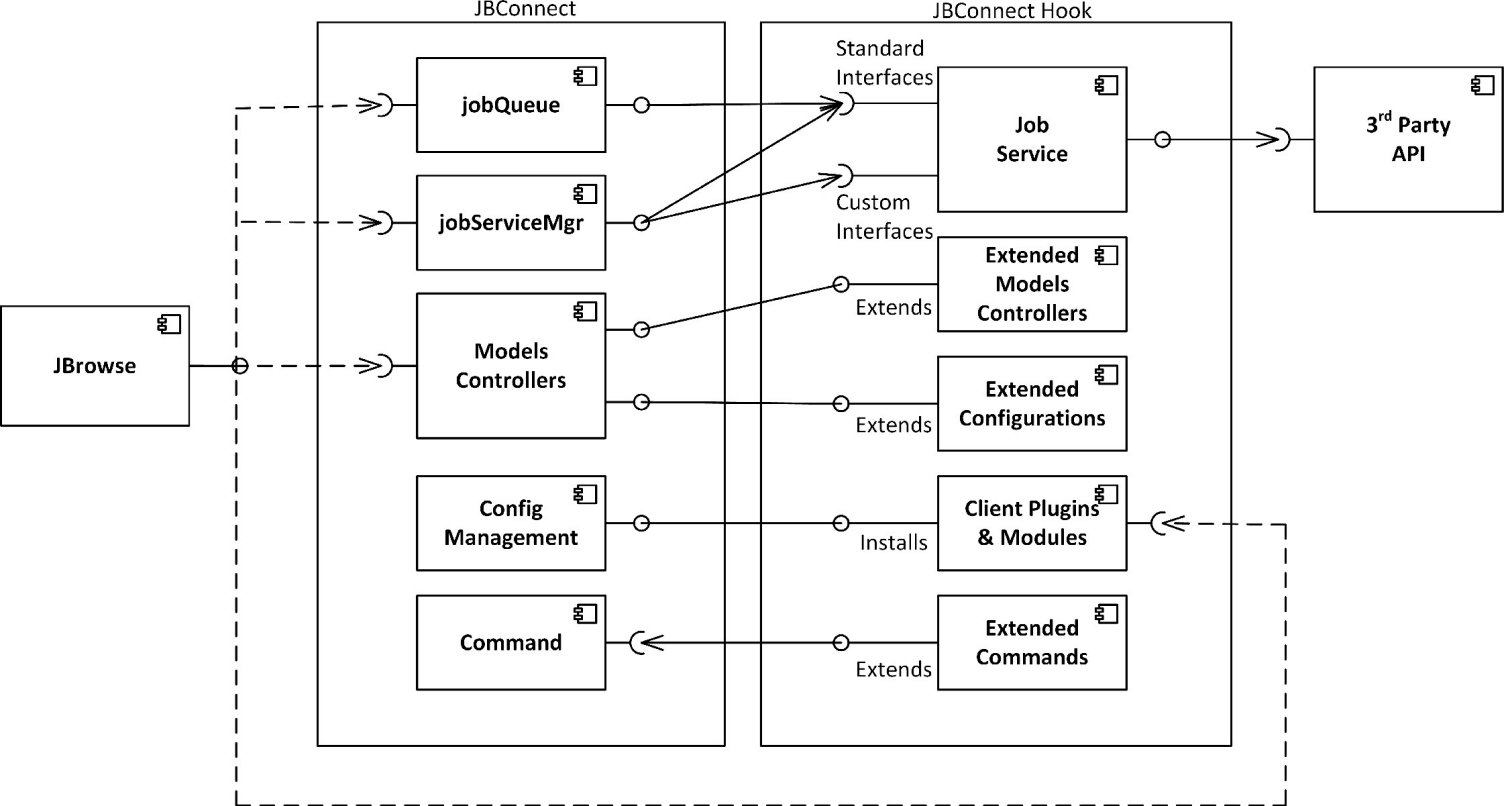
The JBConnect hook framework allows developers to customize most aspects of JBConnect and develop their own workflow extensions.

- Fig 9 shows the JBConnect Hook framework and how JBConnect hooks tie into JBConnect:
- **Job services** are the main mechanism for execution of analysis operations and manages the lifecycle of those operations. The job service can extend the REST API, making it accessible through extended /service/exec REST calls. The client mainly communicates to the job service through the jobServiceMgr module. The job service is the main adapter to provide local or 3rd party service management, such as connections to workflow engines like Galaxy. The jobQueue will launch a Job Service when a queue item is identified to be associated with it.
- **Extended Models and controllers—**Hooks can also extend JBconnect’s models and controllers by way of standard Sails models and controller implementation through the hook’s /api/models, /api/controllers, and services can be extended via the /api/services directory. In the JBlast example, the Job Services (localBlastService and galaxyBlastService) reside in the /api/services directory of the JBlast hook.
- **Extended Configurations—**Hook configurations applied in globals.js are aggregated by JBConnect framework.
- **Client Plugins/Modules**—Plugins include client code that is integrated with the server, along with dependency modules for the plugin. For example, the JBlast plugin, which is deployed with JBlast hook, provides the interface to handle the BLAST filter panels as seen in Fig 2 and Fig 4, as well as the BLAST Feature Details as seen in Fig 3. Plugins are injected into JBrowse by the configuration management module.
- **Extended commands** are extension code that extends the function of JBConnect’s jbutil command which is used for debugging and configurations. An example of this is the jbutil--setupdata command, that sets up some demonstration tracks.

A more detailed description of JBConnect hooks can be found in the online documentation at the URL https://jbconnect.readthedocs.io/en/latest/hooks.html

A tutorial on creating a job service from scratch is provided, illustrating required methods for job execution, is available at the URL https://jbconnect.readthedocs.io/en/latest/tutorials.html

#### The JBClient plugin mediates communication between the JBrowse client and the JBConnect server

JBClient is a client-side plugin for JBrowse that extends the JBrowse graphical user interface and communicates with the JBConnect web services. The JBClient plugin is automatically injected by JBConnect when the server is started. It extends the JBrowse user interface with the login panel and the job queue panel (Fig 1F), and automatically responds to new or modify track events by updating the associated JBrowse views.

#### JBlast demonstrates Galaxy-hosted workflows

As we have described JBlast’s stand-alone BLAST processing (see Fig 5), here we illustrate JBConnect’s ability to manage a third party workflow service, Galaxy, via the Galaxy API. As proof of concept, we have implemented galaxyBlastService job service as part of the JBlast hook.

**Fig 10:**
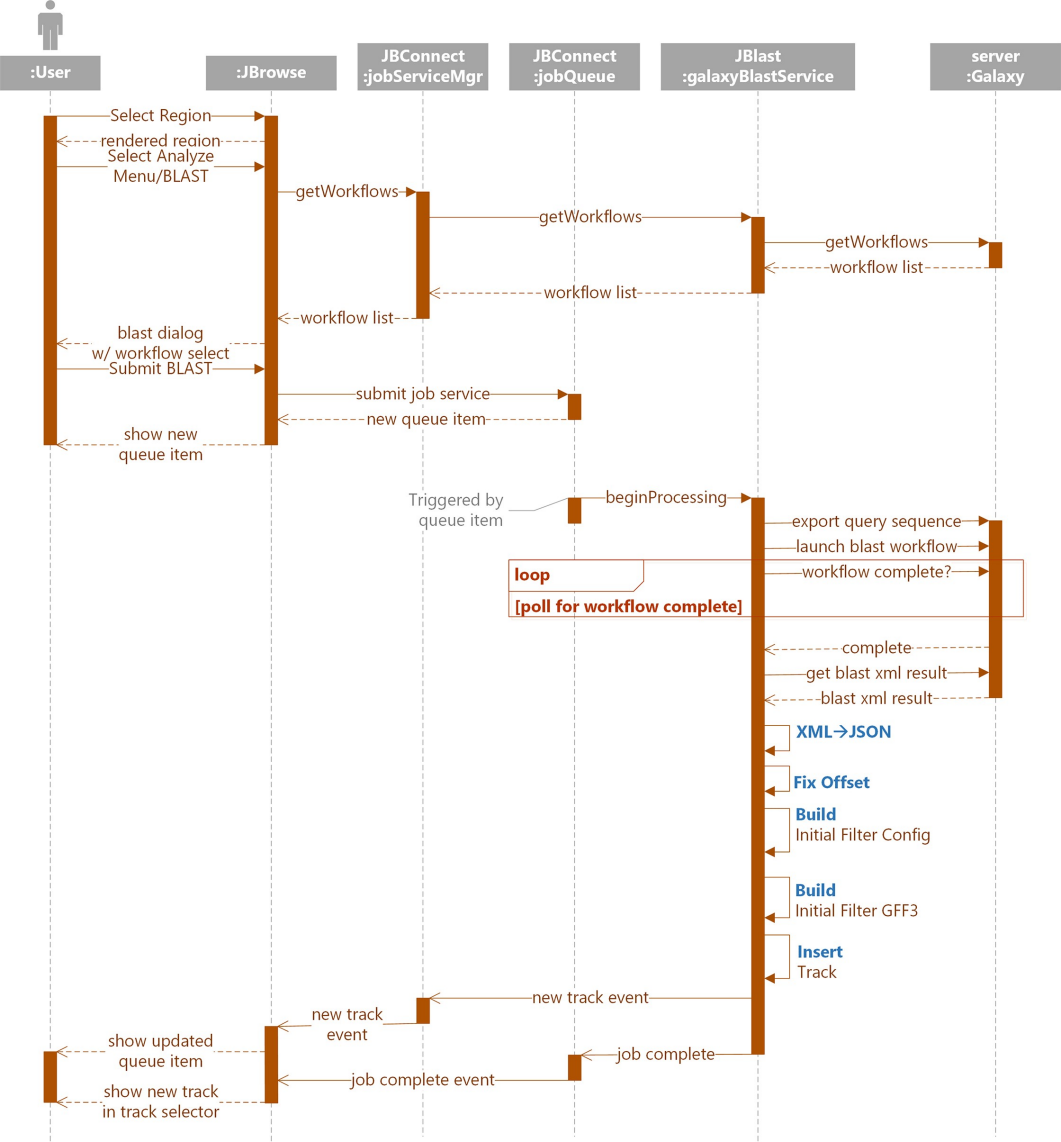
Running a BLAST job using the Galaxy back-end is similar to running BLAST as a standalone service from the user perspective, but involves the galaxyBlastService module that interfaces the Galaxy. galaxyBlastService pre-processes the data before sending it to Galaxy and post-processes the results of the Galaxy workflow run.

Fig 10 illustrates the primary components of the Galaxy BLAST process interaction between JBrowse client, JBConnect server and Galaxy. The components are as shown in Fig 5 and Fig 7, with the following additional notes:

- The jobService control provides the primary interface to the JBlast:galaxyBlastService
  ○ Queue hosts the job queue API
  ○ JBlast:galaxyBlastService manages the life-cycle of a Galaxy job and is the adapter to communicating with Galaxy server.
- The Galaxy server must also be installed for Galaxy-hosted services to run.

JBlast analysis involves coordination between the JBrowse client and a Galaxy instance with a BLAST database, mediated by the JBrowse server. The following is a brief description of each step in the process of launching a BLAST job through its completion of a basic BLAST case:

1. The job submission cycle begins with the user selecting the desired region by highlighting the region with JBrowse. This becomes the query sequence. (See Fig 1B)
2. User selects the BLAST highlighted region from the Analyze menu. (See Fig 1C).
  - JBrowse calls getWorkflow from jobServiceMgr,
  - Which in turn calls getWorkflow from galaxyBlastService,
  - Which acquires the workflow list from Galaxy
  - The Process BLAST dialog then appears. (See Fig 1D) If there is only one workflow, it will automatically be selected and the workflow selection option will not appear.
3. User selects Submit (Fig 1D).
4. A job structure and the query sequence are submitted to the jobQueue.
5. The job queue panel (Fig 1F) will appear showing the submitted job. This completes the job submission cycle.
6. A monitor in the jobQueue detects the queue item then calls beginProcessing in galaxyBlastService. This triggers galaxyBlastService’s execution phase:
  - It exports the query sequence and
  - Launches the selected galaxy workflow.
  - galaxyBlastService now monitors the Galaxy workflow for completion.
  - Upon completion
  - A resulting BLAST XML file is imported from Galaxy
  - The XML is converted to JSON
  - Offsets are adjusted for the resulting hits
  - It builds an initial filter config file for the resulting hits
  - It builds an initial GFF3 file and
  - Inserts the newly created GFF3 as a track in JBrowse
7. A new track event is propagated to JBrowse and the resulting track displayed (See Figs 2A and 4A).
8. As the job completes, the completion event is propagated to the job queue and then to the JBrowse client.

Use of the Galaxy job service requires setting up Galaxy with NCBI blast tools from the Galaxy toolshed and setting up a BLAST workflow within Galaxy that will export results in the XML format consumed by JBlast. The job service communicates with Galaxy through the Galaxy API, for which an API key must be generated from the Galaxy user interface.

Both galaxyBlastService and localBlastService set the initial state of the client filter by determining the range of each filterable value, such as for score, e-value, identity, and gaps).

## Results

Case Study: Using JBlast to discover homoeologous regions in a polyploid plant

In order to concretely describe JBlast usage in a real-world example, we will use the demo JBrowse instance with the JBlast plug-in for the Chinese Spring IWGSC (International Wheat Genome Sequencing Consortium) RefSeq v1.0 wheat genome assembly [12]. The demo JBrowse instance can be accessed at a GrainGenes development site at http://graingenes.org:1337/jbrowse/?data=IWGSC. As a simple security feature, users must authenticate; a guest login/password pair (juser/password) are provided for evaluation purposes.

In contrast to a BLAST session performed using command-line or on a customized BLAST interface, JBlast offers immediate integration of the resulting sequence alignments with JBrowse’s annotation visualization capabilities. JBlast results appear as tracks, allowing in-depth exploration of genomic elements and annotations in the proximity of the JBlast hits.

The Chinese Spring wheat genome provides a good case study. It is a hexaploid plant, with six sets of seven chromosomes (2n = 6x = 42). BLAST hits are expected to be distributed across homoeologous chromosomes, which are historically named as A, B, and D [14–17].

This case study is the example used in Fig 1. In the demo instance of JBlast hosted at GrainGenes for Chinese Wheat, the example sequence provided in the dialog box for the “BLAST DNA sequence” (as shown e.g. in Fig 1E) is a truncated 5’ sequence of the TraesCS1A02G160100 gene, located on chromosome 1A, which codes for a ribosome-associated protein according to the Gene Ontology annotations for the gene. When users choose the example as their query sequence, the BLAST job should generate four hits. Setting the sequence identity threshold in the BLAST hit filter to 90% should filter this down to three hits, listed for convenience in the filter, and ordered according to the BLAST e-value of the hit. These hits are on chromosomes 1A, 1B, and 1D. As shown in Fig 4, the BLAST alignment for a given hit can be observed by clicking on the displayed feature corresponding to that hit. Then, by adjusting the zoom level, the user can explore the local landscape of features flanking the putative homeoloci.

In our demonstration, three hits to the 5’ sequence of TraesCS1A02G160100 are found, aligned to the following gene models: TraesCS1A02G160100 (self) on Chromosome 1A (sequence identity: 100%), TraesCS1B02G176500 on Chromosome 1B (sequence identity: 93%), and TraesCS1D02G157300 on Chromosome 1D (sequence identity: 93%). According to the official annotations provided by the IWGSC, these three high-confidence gene models have the following common gene ontology (GO) annotations: GO:0005840 (ribosome), GO:0003735 (structural constituent of ribosome), GO:0008097 (5S rRNA binding), GO:0006412 (translation), GO:0008152 (metabolic process).

These results illustrate how JBlast enables quick identification of gene models of putative homeoloci, which can be further analyzed by comparing and studying the biological information embedded in other JBrowse tracks. This case study demonstrates not only the simplicity and convenience of using JBlast to browse and navigate BLAST hits, but also provides evidence about its usefulness when looking at gene families for any species and for homeoloci in any polyploid.

## Availability

Source code for JBrowse Connect is at https://github.com/gmod/jbconnect

The JBlast hook for JBrowse Connect is at https://github.com/GMOD/jblast-jbconnect-hook

The GrainGenes demo instance is at http://graingenes.org:1337/jbrowse/?data=IWGSC

JBrowse Connect and JBlast tutorials are available at https://jbconnect.readthedocs.io/

## Future directions

JBrowse has previously been integrated with genomics-oriented content-management systems such as Tripal [6], as well as computation services such as Cyverse [18] and bioinformatics pipeline managers such as Maker [19], SeqWare [20], and Galaxy [21]. JBrowse has been implemented as a tool in Galaxy (https://biostar.usegalaxy.org/p/15491/). Galaxy also has its own genome browser, with a streamlined set of features compared to JBrowse, from which it is possible to launch and track Galaxy jobs. A basic “BLAST” button (lacking Galaxy integration or queue monitoring) has been available for some time to biocurators using the genome annotation editor Apollo [4], which is built using JBrowse.JBrowse Connect builds on these previous efforts to offer a general-purpose framework for doing analysis from within the established, richly-functional, and well-supported JBrowse genome browser. The user experience is well-integrated with browsing: the user can maintain focus on biological objects of interest, i.e. genomes and genome annotations, without making unnecessary detours to a separate workflow-management application. The analysis queue is a minimally-invasive popup window accessible from within JBrowse. BLAST results appear immediately within their genomic context, and are instantaneously available for other users of the site to share if the site has been configured so as to allow sharing of BLAST results tracks.

Aside from performing alignments or sequence similarity searches, the JBrowse Connect infrastructure can in principle be used for many common tasks around genomics web development (and content management more generally) such as persistent user uploads and data-sharing, automatic indexing of uploaded files, exposure of more JBrowse functions as server-hosted web services, and policy-based access controls.

JBrowse Connect analyses can all be handled in a manner complementary with the local policies and server software for authentication, access control, and content management. Security is naturally an issue, and exposure of any form of code to user-initiated queries naturally compromises security, relative to a static-site client-only application such as JBrowse. The additional compartmentalization and layering of the JBrowse Connect architecture, relative to the Galaxy or simple server-hosted BLAST model (as shown in Figs 5, 7 and 10), can afford some security protections. Nevertheless, some tradeoff between security and functionality is inevitable when allowing analysis of user-uploaded data, and this must be borne in mind when deciding whether to expose such services. In this context, it is worth noting that JBrowse Connect is an entirely optional extension to JBrowse. For site administrators who prefer the strong security and light CPU footprint of static hosting, JBrowse is and will remain usable without the extra server component. Further, JBrowse Connect is backward-compatible: if a JBrowse instance is in place, but evolving requirements have revealed a need for a more robust server component (whether it be to notify users of new tracks in real time, to allow them to initiate analyses or workflows from the browser, or for other reasons), then JBrowse Connect can be configured to work with the existing JBrowse instance with minimal disruption. JBrowse Connect uses the same filesystem structure and file formats as JBrowse, so that indexing or data files do not need to be regenerated or modified in order to install the server component.

As bioinformatics web applications develop, genomes and genome annotations are arguably one of the most materially intuitive data structures we have. A transcript is linear not just because it represents a conceptual ordering that is isomorphic to a linear graph, but because it represents a real-world object that has the topology of a line. Thus, it also represents a natural “social object” [22] for scientific collaboration. In this context, it seems likely that more and more applications will coalesce around the genome browser, either as a chassis or as a common component. The JBrowse Connect system aims to accelerate this process by implementing many of the common tasks required to turn a modern static web experience into a dynamic, collaborative one.

### Tutorials

Two tutorials are provided to further illustrate how to build modules that utilize JBConnect. Fully functional sample code is provided as well. The first tutorial demonstrates how to utilize localCommonService Job Service to create a common workflow that is similar to the one used in JBPrimer3 and JBlast. The second tutorial shows how to build a stand-alone Job Service.

*Creating JBConnect Hook*: https://jbconnect.readthedocs.io/en/latest/tutorials.html

*Creating a Stand-Alone Job Service for local workflow processing*: https://jbconnect.readthedocs.io/en/latest/tutorials.html#creating-a-stand-alone-job-service-for-local-workflow-processing
